# Case Report: A Case of *Sarocladium strictum* Meningoencephalitis in an Immunocompetent Patient After Invasive Operation

**DOI:** 10.3389/fmed.2021.762763

**Published:** 2021-11-10

**Authors:** Yue Cui, Jiali Meng, Jing Zhang, Lin Wang, Haihan Yan, Han Xia, Jingrong Cao, Liyong Wu

**Affiliations:** ^1^Department of Neurology, Xuanwu Hospital, Capital Medical University, Beijing, China; ^2^Department of Scientific Affairs, Hugobiotech Co., Ltd., Beijing, China; ^3^Department of Clinical Laboratory, Xuanwu Hospital, Capital Medical University, Beijing, China

**Keywords:** meningoencephalitis, *Sarocladium strictum*, metagenomic next-generation sequencing, fungal infection, treatment

## Abstract

As an opportunistic phytopathogen, *Sarocladium strictum* has only been shown to cause neurological disease in immunocompromised patients, where antifungal therapy was not effective. We report a case of *Sarocladium strictum* meningoencephalitis in an apparently immunocompetent young woman who presented with severe headache and slight fever after undergoing transnasal endoscopic repair of cerebrospinal fluid rhinorrhea. Chronic sinusitis and suspicious intracranial fungal lesions were observed on enhanced magnetic resonance imaging (MRI). Both culture and metagenomic next-generation sequencing of her cerebrospinal fluid were positive for *Sarocladium strictum*. After local debridement, treatment with amphotericin B plus voriconazole and Ommaya reservoir implantation, the patient improved significantly. Unfortunately, her symptoms worsened again despite plenty of antifungal therapy for a month.

## Introduction

*Sarocladium*, under the order Hypocreales, has traditionally been considered an opportunistic phytopathogen, and only a very few species of this pathogen can infect patients with predisposing conditions (1). *Sarocladium strictum*, initially isolated from *Triticum aestivum*, has increasingly been recognized as a clinically important species and was reallocated from *Acremonium* based on rDNA sequences in 2011 by Summerbell et al. (1, 2). Although there have been some reports of *S. strictum* infections in humans, most of them were derived from skin or blood, and the brain is considered one of the least susceptible organs (3). Until now, *S. strictum* meningoencephalitis has been reported only in three immunodeficient patients, all of whom died within a short time (4). Here, we report a case of an apparently immunocompetent young woman who presented with severe headache and slight fever after undergoing a transnasal endoscopic repair of cerebrospinal fluid (CSF) rhinorrhea. Both culture and metagenomic next-generation sequencing of her CSF were positive for *S. strictum*.

## Case Description

On March 8, 2021, a 51-year-old woman who worked as a peasant farmer in Inner Mongolia, China, sought treatment at Xuanwu Hospital, Beijing, for continuous fever and headache after undergoing a transnasal endoscopic repair for spontaneous rhinorrhea. The patient was devoted to the autumn harvest before the onset of symptoms and reported no travel history or animals contact. None of her family members or neighbors showed similar symptoms or other signs of infection at the same time. Her preexisting condition was a history of penicillin allergy.

Three months before admission to Xuanwu Hospital, the patient experienced spontaneous cerebrospinal rhinorrhea and underwent a transnasal endoscopic repair surgery in a regional hospital, after which she began to develop night fever with intermittent headache. Meanwhile, multiple enhanced sinus magnetic resonance imaging (MRI) revealed increasingly severe bilateral nasosinusitis. In addition, her intracranial pressure was >300 mmH_2_O, and her CSF examination showed a high white blood cell (WBC) count with predominance of mononuclear cell (~70%) and high protein (95.40 g/L). The CSF-to-serum glucose ratio decreased to 0.53. Based on this evidence, she was diagnosed with meningoencephalitis and treated with a full course of intravenous meropenem (2.0 g per 8 h) and vancomycin (0.5 g per 6 h). However, her condition continued to deteriorate. Despite switching to anti-infective treatment with meropenem (as before), linezolid (1.0 mg/kg per 12 h intravenously), and fluconazole (400 mg per day orally), the patient's headaches persisted. Moreover, she began to present with blurred vision, epileptic seizures, and hallucination. In addition, her intracranial pressure rose higher than before (380 mmH_2_O).

**Figure 1:**
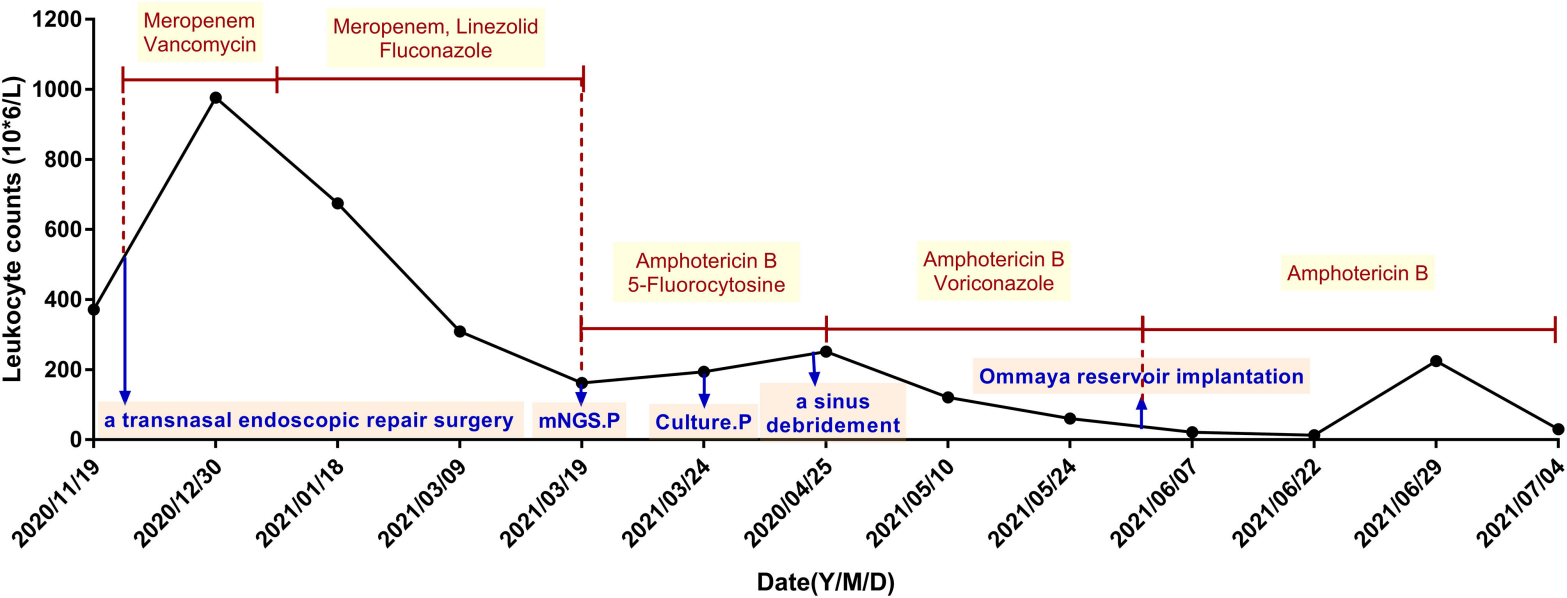
The treatment procedures and corresponding leukocyte counts in the patient's cerebrospinal fluid. Monocytes were predominant among CSF white cells; they had a proportion of approximately between 60 and 90%. The CSF protein level was also elevated to 95.00–125.20 mg/dl. mNGS.P, metagenomic next-generation sequencing of cerebrospinal fluid detected *Sarocladium strictum* positive; Culture.P, *Sarocladium strictum* was positive in cerebrospinal fluid culture.

At admission of Xuanwu Hospital, the main complaint of the patient was persistently severe headache, and physical examination revealed obvious meningeal irritation and eye movement disorders. Bilateral papilledema and abnormal signals at the right basal ganglia region were also found. She was also tested for HIV, syphilis, cancer, cellular and humoral immunity via flow cytometry, and immunoglobulins. All these tests were negative. Metagenomic next-generation sequencing (mNGS) and culture were done on collected CSF specimens 5 times during hospitalization. The infection of *S. strictum* was verified through culture and mNGS of CSF samples on March 24th and March 19^th^, respectively. Thereafter, the patient was treated with amphotericin B (1.0 mg per day originally and increased by 2 mg every other day for 7 days, then increased by 5 mg per week, intravenously) and 5-fluorocytosine (6.0 g per day orally in four divided doses), instead of antibacterial drugs. Moreover, she underwent sinus debridement, but the effect was not ideal. It was only after changing the antifungal therapy to amphotericin B plus voriconazole (4.0 mg/kg per 12 h intravenously) that her headache and epileptic seizures got relieved than before with normal body temperature. The WBC count in her CSF also reduced gradually (Figure 1). At the time of discharge, intravenous amphotericin B dosage had reached 25 mg per day and the total dose was 742 mg.

Soon afterwards, the patient accepted Ommaya reservoir implantation to extract CSF and inject amphotericin B (0.2 mg per day) in a regional hospital, with intravenous amphotericin B (20.0 mg per day), which was extremely effective against intracranial hypertension. Her headache was relieved further, with no more epileptic seizures and hallucination, and her eyesight was partially restored. Unfortunately, although under a plenty of antifungal therapy, the patient's condition worsened again within a month. On June 29, 2021, the patient began to present with worsening headache and blurred vision, with unstable standing and poor coordination of fine movements. The WBC count in her CSF was also elevated again, and MRI revealed multiple new punctured abnormal signals in her bilateral cerebellum. After the addition of itraconazole orally, the WBC count in her CSF decreased, but her symptoms remained unrelieved. Eventually, the patient requested to take itraconazole orally (0.2 g per day orally in two divided doses) at home, and she still suffered from headache and blurred vision on September 29, 2021. The clinical case was approved by the Ethics Committee of the Xuanwu Hospital of Capital Medical University, China. Written informed consent was obtained from all the patients.

**Figure 2:**
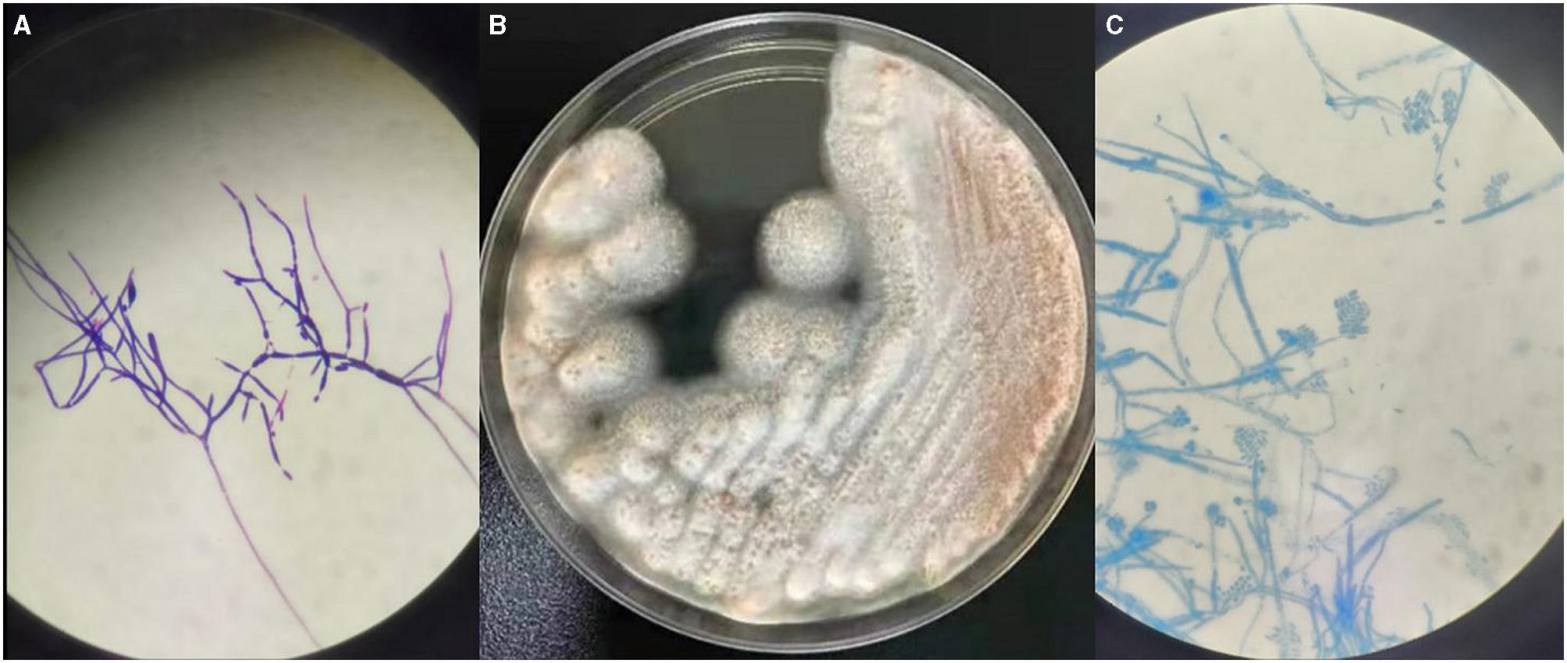
**(A)** The fungal hyphae by Gram stain (×100). **(B)** The colony on Sabouraud's agar plates after 2d at 28°C. **(C)** Micromorphology of a colony of *Sarocladium strictum* (lactophenol cotton blue preparation, ×100).

In our study, *S. strictum* was isolated and identified at the Clinical Laboratory of Xuanwu Hospital. BD BACTECTM FX (PEDS plus) was used for both blood and CSF cultures, but only CSF was positive on the third day (3d2h58m) of incubation. Floccus grew in a positive culture bottle of CSF, and fungal hyphae could be visualized on Gram staining (Figure 2A). Positive cultures were transferred onto sheep blood agar plates at 35°C and Sabouraud's glucose agar plates at 28°C. White tufted colonies with a pale salmon pink-colored base were observed (Figure 2B). Lactophenol cotton blue preparations from the colonies revealed abundant, small, cylindrical conidia that were produced from the phialide tips of long, slender, lateral hyphae (Figure 2C). These findings are in accordance with the morphological characteristics of *S. strictum* which could not be identified through Matrix-Assisted Laser Desorption Ionization Time of Flight Mass Spectrometry (MALDI-TOF MS, Bruker) and was finally identified by ITS sequencing. It had 99% homology with known strains of *S. strictum* in GenBank.

**Figure 3:**
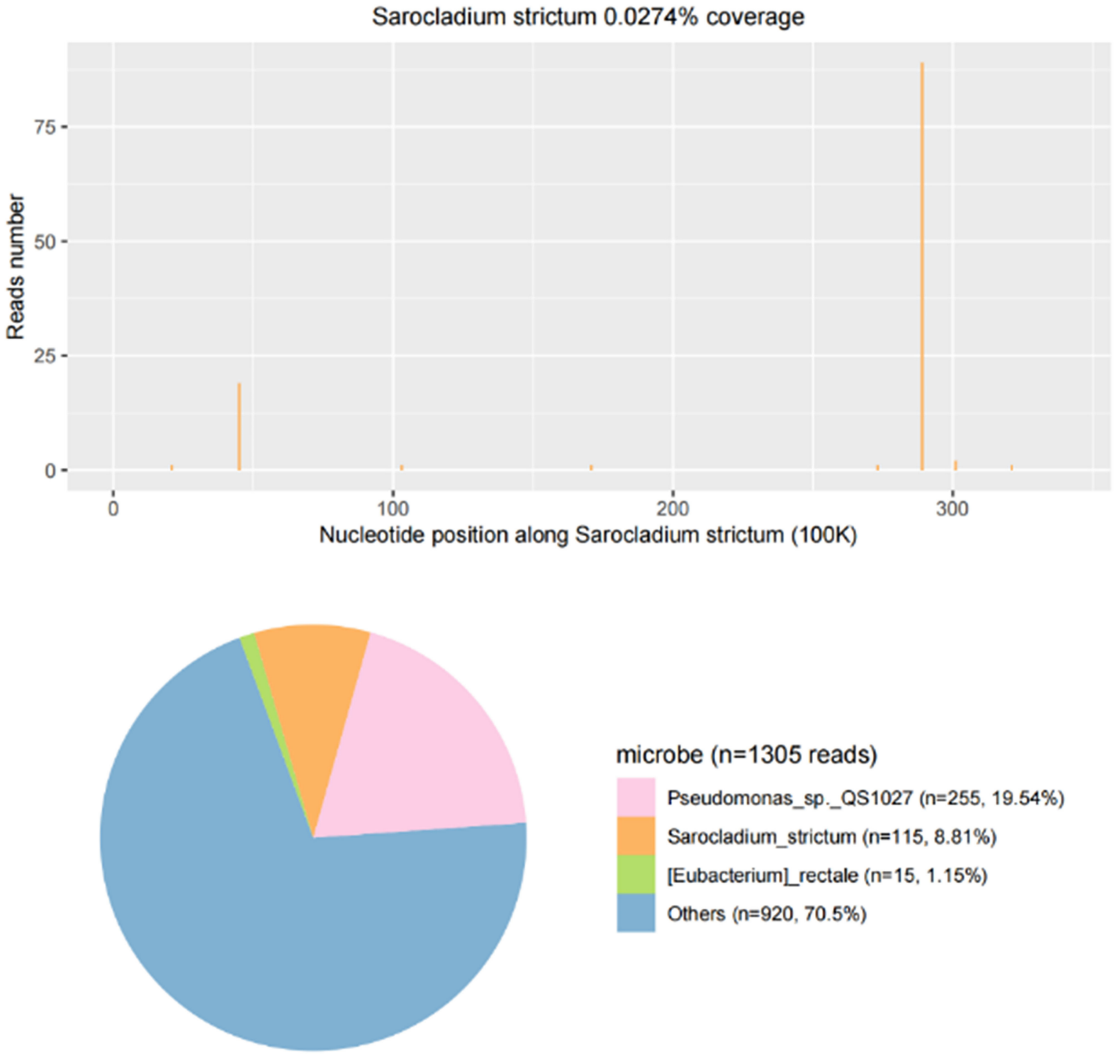
The coverage and abundance of *Sarocladium strictum* detected by mNGS on March 19, 2021.

CSF samples were transferred to dry ice for PACEseq mNGS detection (Hugobiotech, Beijing, China). DNA was extracted using the QIAamp DNA Micro Kit (Qiagen). QIAseq™ Ultralow Input Library Kit (Illumina) was used to construct the DNA libraries. After purification, the quality of the libraries was analyzed using a Qubit (Thermo Fisher) and Agilent 2100 Bioanalyzer (Agilent Technologies). The NextSeq 550 platform (Illumina) was used to sequence all the DNA reads in the libraries. After sequencing, short, low-quality, and low-complexity reads were removed from the raw data. Human DNA was also filtered out according to the human reference database. The clean reads were aligned to the microbial genome database (NCBI; ftp://ftp.ncbi.nlm.nih.gov/genomes) to identify all probable pathogens. Moreover, mNGS revealed *S. strictum* in the CSF samples on the second day after lumbar puncture, with coverage and abundance shown in Figure 3. Furthermore, detected reads are shown in Supplement File 1.

## Discussion

The first case of *S. strictum* infection in the brain was reported in 1987 in a 9-year-old boy who received long-term corticosteroid treatment for Landry-Guillain-Barré syndrome with subacute *S. strictum* meningitis (5). In 1993, *S. strictum* was cultured from the brain abscess of a 60-year-old woman who was treated with antineoplastic chemotherapy for bronchoalveolar carcinoma before her death (6). Unfortunately, due to the age of the reports, their diagnostic accuracy was uncertain, and their therapeutic process was unavailable. The only detailed case reported in 2003 was that of a neonate born at 27 weeks of gestation who was diagnosed with *S. strictum* infection through blood and CSF cultures, as well as autopsy, with no response to therapy with amphotericin B plus fluconazole (7). In contrast to previous reports, *S. strictum* infection of the brain was only detected in critical patients with immunodeficiency. We reported an immunocompetent young woman diagnosed with *S. strictum* meningoencephalitis using both morphological and molecular methods.

*Pseudomonas* sp.QS1027 (255 specific reads) were also caught by mNGS of CSF. However, this microbe was previously discovered in *Dictyostelium discoideum* fruiting bodies and always found in the background of mNGS, including negative controls due to the use of multiple reagent buffers (8). This indicated that *Pseudomonas* sp.QS1027 was more likely a reagent engineering bacterium (9, 10). In addition, there is no clinical evidence of *Pseudomonas* sp.QS1027 to cause infections in human, and the patient's clinical characteristics did not match those of *Pseudomonas* infection (11). Thus, this bacterium was not considered as a potential causative agent in this case.

Before the onset of meningoencephalitis, the patient underwent transnasal endoscopic repair due to progressive nasosinusitis. Several blood cultures were negative during the entire disease course, which strongly excluded the possibility of hematogenous spread and supported that the intracranial infection was caused by the dissemination of local infection after invasive operation. This study suggested that in addition to patients with predisposing conditions, healthy humans who have undergone surgery or other invasive operations.

Although there have been many reports on the susceptibility of *S. strictum*, with fluconazole and 5-fluorocytosine being considered ineffective in *S. strictum* infection, evidence regarding the effectiveness of amphotericin B has been greatly conflicting (4, 12). As an effective agent against most fungi, amphotericin B is often used as the first choice for *S. strictum* treatment and has shown promising results in cases of pulmonary infection (13). However, the patient had no response to amphotericin B plus 5-fluorocytosine in this case, and her CSF leukocyte count increased. Her symptoms did not improve until 5-fluorocytosine was switched to voriconazole. Previously, an *in vitro* study showed the high diversity and variable susceptibility of *Sarocladium* species. It was shown that *S. strictum* was susceptible to voriconazole and terbinafine but was resistant to amphotericin B (4). Although it could not be ruled out that amphotericin B might not be effective at initial doses, we believe that voriconazole also plays a critical role in the treatment of *S. strictum* meningoencephalitis. After discharged from Xuanwu hospital, the patient was treated with intravenous amphotericin B and Ommaya reservoir. The treatment was effective at first in relieving symptoms of cranial hypertension but turned ineffective before long, suggesting that Ommaya reservoir implantation may be a good strategy to control the symptoms of intracranial infection. However, appropriate drug selection remains a top priority in the treatment of intracranial fungal infections.

This case suggests the possibility of *S. strictum* meningoencephalitis in an immunocompetent patient and provides clinical evidence for its effective treatment. Since different species of *Sarocladium* have varying susceptibility to antifungal agents, mNGS, which identifies pathogens early and distinguishes species accurately prior to culture, is of great significance in guiding clinical medication.

## Data Availability Statement

The datasets presented in this study can be found in online repositories. The names of the repository/repositories and accession number(s) can be found below: National Genomics Data Center, accession no: PRJCA006827.

## Ethics Statement

The studies involving human participants were reviewed and approved by the Ethics Committees of the Xuanwu Hospital of Capital Medical University, China. Written informed consent was obtained from the patient.

## Author Contributions

YC provided the initial and subsequent draft of the submission. JM, JZ, LWa, and HY provided patient details and time activity curves. HX and JC provided images and microbiological analysis. LWu helped to create subsequent drafts of the submissions and coordinated the editing process. All authors contributed to the article and approved the submitted version.

## Funding

This work was supported by grants from the Ministry of Science and Technology of China (No. 2019YFC0118600) to LWu, National Key Research and Development Program of China Research on the Precision Diagnosis, Treatment, and Integrated Prevention, Control for the Elderly with Common Infectious Disease (2020YFC2005403) to Professor Yan Zhang, and the Xuanwu Hospital Teaching Reform Foundation (2020XWJXGG-09) to JC.

## Conflict of Interest

HX was employed by the company Hugobiotech Co., Ltd. The remaining authors declare that the research was conducted in the absence of any commercial or financial relationships that could be construed as a potential conflict of interest.

## Publisher's Note

All claims expressed in this article are solely those of the authors and do not necessarily represent those of their affiliated organizations, or those of the publisher, the editors and the reviewers. Any product that may be evaluated in this article, or claim that may be made by its manufacturer, is not guaranteed or endorsed by the publisher.
